# 2482. Cross Validation of Surgical Procedure Data in National Healthcare Safety Network vs Hospital Discharge Data

**DOI:** 10.1093/ofid/ofad500.2100

**Published:** 2023-11-27

**Authors:** Yoran (Lana) Sato, Andrea M Parriott, N Neely Kazerouni, Erin Epson

**Affiliations:** California Department of Public Health, Dublin, California; California Department of Public Health, Dublin, California; CA Dept of Public Health, Sacramento, California; California Department of Public Health, Dublin, California

## Abstract

**Background:**

California acute care hospitals (ACH) are required to report data on 28 types of inpatient surgical procedures via the National Healthcare Safety Network (NHSN). ACH also report discharge data, including procedure data, to the California Department of Healthcare Access and Information (HCAI). We compared the number of adult (age >=18) procedures reported in NHSN versus HCAI in 2019 for cardiac surgery (CARD), coronary bypass with chest and donor incisions (CBGB), hip prosthesis (HPRO), kidney transplant (KTP), and rectal surgery (REC).

**Methods:**

For both systems, we aggregated procedure counts to the facility level and matched individual facilities. HCAI ICD-10 codes were mapped to NHSN procedure codes. Because NHSN counts multiple procedures that map to the same procedure code done via the same incision as one procedure and HCAI does not specify how many procedures were done per incision, CARD, CBGB, and REC were counted only once per procedure code per patient per day. HPRO and KTP were counted up to twice per patient per day if ICD-10 codes for both right and left side surgeries were present. ANOVA was used to compare the means of log standardized infection ratio (SIR) by NHSN to HCAI procedure count ratio as categories of less than 0.9, between 0.9 and 1.1, and more than 1.1.

**Results:**

Three hundred eleven facilities reported 82,847 procedures to NHSN and 300 facilities reported 91,961 procedures to HCAI. Median and interquartile interval for the facility-specific ratio of NHSN to HCAI procedures among facilities that reported procedures in both systems are displayed in Table 1. ANOVA failed to detect any difference in mean of log SIR by different NHSN to HCAI procedure count ratio at α of 0.05.

**Figure:**
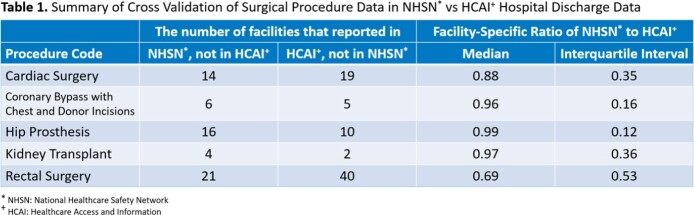

**Conclusion:**

For all procedure types, there were more procedures documented in HCAI than NHSN, but the ratios of NHSN procedures to HCAI procedures varied widely by procedure type. However, difference in ratio was not associated with SIR. Ability to compare procedure counts was limited by inability to ascertain operating room deaths (which are not counted in NHSN) and if procedures were done using the same incision in HCAI. Further analysis is recommended since SSI denominator quality is crucial to the accuracy of SIR, which informs many analyses and interventions.

**Disclosures:**

**All Authors**: No reported disclosures

